# Saliva as a potential matrix for evaluating pharmacologically active dolutegravir concentration in plasma

**DOI:** 10.1371/journal.pone.0246994

**Published:** 2021-02-18

**Authors:** Eiko Yamada, Ritsuo Takagi, Hiroshi Moro, Koji Sudo, Shingo Kato

**Affiliations:** 1 Division of Oral and Maxillofacial Surgery, Faculty of Dentistry & Graduate School of Medical and Dental Sciences, Niigata University, Niigata, Japan; 2 Department of Respiratory Medicine and Infectious Diseases, Niigata University Graduate School of Medical and Dental Sciences, Niigata, Japan; 3 Hanah MediTech Co. Ltd., Tokyo, Japan; 4 Department of Microbiology and Immunology, Keio University School of Medicine, Tokyo, Japan; University of Porto, PORTUGAL

## Abstract

Therapeutic drug monitoring (TDM) is used in certain clinically selected cases and in research settings to optimize the response to antiretroviral therapy. Plasma of blood is commonly used for TDM, but blood sampling is invasive and at risk for transmission of infectious agents. On the other hand, saliva sampling is noninvasive, safe, cheap, and easily performed compared to blood. Dolutegravir (DTG) is now widely prescribed as a key component of antiretroviral therapy for HIV infection. In this study, we examined the relationship between DTG concentrations in plasma and saliva of treated patients to explore the possibility of using saliva as an alternative body fluid of TDM. A total of 17 pairs of blood and saliva samples were obtained from 15 consented HIV-1-infected subjects treated with DTG containing regimens for more than one month. Both blood and saliva samples were collected within 1 h of each other. Drug concentrations were determined by liquid chromatography-tandem mass spectrometry using DTG-d5 as an internal standard. The LLOQ was 0.5 ng/mL. The calibration curves were prepared with pooled plasma or saliva containing DTG in a range of 0.5–100 ng/mL with precision of <14.4% and accuracy within ±14.7%. The DTG concentrations in the plasma and saliva were significantly correlated (Pearson’s correlation coefficient *r* = 0.76, *p* < 0.001). The median ratio of the drug concentration in saliva to those in plasma was 0.0056, which is close to the rate of non-protein-bound DTG in plasma (0.70%), suggesting that only free DTG in plasma is transported to the salivary glands and secreted into saliva. The present study demonstrates that DTG concentration in saliva reflects the pharmacologically active drug concentration in plasma and may provide an easily accessible alternative for monitoring effective antiretroviral treatment.

## 1. Introduction

Therapeutic drug monitoring (TDM) is used in certain clinically selected cases and in research settings to optimize the response to antiretroviral therapy (ART) [1]. However, frequent blood sampling may pose a risk of viral transmission to medical staff and cause severe distress to patients. In contrast, saliva sampling is noninvasive, safe, and, cheap; it can be performed at home by the patient, even if it is a child [2, 3]. We have previously shown that saliva can be used as an alternative of blood plasma for four antiretroviral drugs (abacavir, tenofovir, darunavir, and raltegravir) [4]. In recent ART, dolutegravir (DTG) is widely used as a key drug because of its potent activity, lower reduced toxicity, a high genetic barrier to resistance, and advantageous pharmacokinetics [5].

Several liquid chromatography-tandem mass spectrometry (LC-MS/MS) methods have been described for the quantification of DTG in plasma [6–9]. However, there has been no method reported for the quantification of DTG in saliva. Therefore, we developed and validated a common LC-MS/MS method for both plasma and saliva.

The aim of this study was to evaluate the possibility of using saliva as an alternative to plasma for TDM of DTG by examining the relationship between DTG concentrations in plasma and saliva of treated patients determined by the LC/MS-MS method presented here.

## 2. Materials and methods

### 2.1 Ethics statement

Written informed consent was obtained from all participants. The study was approved by the Ethical Committee of Niigata University (2017–0181).

### 2.2 Participants

A total of 17 pairs of blood and saliva samples were obtained from 15 HIV-1-infected patients (11 men and 4 women) who were treated with the regimen containing 50 mg of DTG once a day for more than one month with good adherence evidenced by high virological suppression. Both blood and saliva samples were collected within 1 h of each other and stored at −20°C before use. The intervals from the dose to the sample collection varied from 2 h to 24 h. No patients had abnormal liver or kidney function.

### 2.3 Chemicals

DTG and DTG-d5 were purchased from Cayman Chemical (Ann Arbor, MI, USA). Analytical-grade methanol and formic acid were obtained from Nakalai Tesque (Kyoto, Japan). Water was deionized and osmosed using Milli-Q system (Merck Millipore, Burlington, MA, USA). Plasma (citrate) was purchased from Kojin Bio (Saitama, Japan) and saliva was obtained as described below from two healthy individuals and combined. These plasma and saliva were used as negative controls.

### 2.4 Standard solutions

Stock solutions (1 mg/mL) of standard and internal standard (IS) were prepared by dissolving DTG and DTG-d5 in methanol, respectively. They are stored at −20°C and returned to room temperature before use.

### 2.5 Sample preparation

Blood samples were collected into a 10 mL EDTA tube and then centrifuged at 3,000 rpm for 8 min at 25°C to obtain plasma. Saliva samples (2–3 mL) were collected without any stimulation into a 50 mL tube by expectoration after gargling with water. Both plasma and saliva samples were stored at −80°C and thawed at room temperature before use. The plasma samples were diluted 1:100 with phosphate buffer saline before measurement because they had about 100-fold higher DTG concentration ranges than saliva samples and diluted samples permitted to minimize contamination and loss of sensitivity. Plasma and saliva samples (36 μL) were spiked with 4 μL of DTG-d5 (23.8 ng/mL) that correspond to 95.2 pg and each mixed 1:1 with acetonitrile to precipitate the proteins [9] and centrifuged at 12,000 rpm for 3 min at 25 °C to remove precipitated material. Then, 40 μL of supernatant were dried in a vacuum centrifuge and dissolved in 20 μL of 5 mM formic acid-25% (v/v) ethanol.

### 2.6 LC-MS/MS

DTG concentrations were determined using a LC-MS/MS, LCMS-8030 system (Shimadzu, Kyoto, Japan). Analytes were separated isocratically on a reverse phase C_18_ column (1.5 × 50 mm, 5 μm, Inertsil^®^ ODS-3, GL Science, Tokyo, Japan) with 5 mM formic acid-25% (v/v) ethanol at a flow rate of 0.2 mL/min. The column temperature was maintained at 40°C and the sample plate was kept at 4°C. The analytes were detected using positive electrospray ionization mode with multiple reaction monitoring (MRM). The injection volume was 1 μL, and the total analysis time for each sample was 6 min. Argon was used as the nebulizer and desolvation gas. The gas temperature, nebulizing gas flow, drying gas flow, capillary voltage, and collision energy were set at 250°C, 3.0 L/min, 15.0 L/min, 6000 V, and −30 V, respectively. Precursor to product mass transitions for DTG and DTG-d5 were 420/127 and 420/132 (*m*/*z*), respectively. LabSolutions software (Shimadzu, Tokyo, Japan) was used for system control and data analysis.

### 2.7 Calibration curve

Calibration curves were obtained separately for plasma and saliva using one blank sample, one zero sample (blank matrix plus IS), and standard samples prepared at eight concentrations (0.5, 1, 2, 5, 10, 20, 50, and 100 ng/mL). DTG-d5 (4 μL, corresponding to 95.2 pg) was added as IS. The ratio of peak area to IS peak area was analyzed using linear regression with a weighting factor of 1/(nominal concentration)^2^. The lower limit of quantification (LLOQ) was estimated to meet the condition of the analyte peak area being at least 5 times of the corresponding blank area, accuracy was within ±20%, and precision was <20%. At least five of the eight non-zero samples were required to meet the criterion that the back-calculated values of the standards were within ±15% of the nominal concentration, except at LLOQ, for which ±20% was acceptable. Standards not meeting these criteria were excluded from the curve calculation.

### 2.8 Method validation

Validation of the assay was conducted according to Guidance for Industry, Bioanalytical Method Validation published by the Food and Drug Administration [10]. Evaluations of selectivity, accuracy, and precision were conducted as described previously [11]. Selectivity with respect to endogenous and exogenous components in the matrix (plasma or saliva) was evaluated using six blank samples from commercial plasma treated without antiretroviral agents. The blank samples were spiked with the analyte at 0.5 ng/mL and assayed in triplicate. The accuracy and precision of the method were assessed in five replicates at five concentrations: LLOQ (0.5 ng/mL), upper limit of quantification (ULOQ, 100 ng/mL), low quality control (QC) (1 ng/mL), middle QC (5 ng/mL), and high QC (50 ng/mL). The accuracy was calculated as the percent deviation between the nominal and mean values; the precision was calculated as the coefficient of variation (CV). The acceptance criterion for accuracy was within ±15%, except at LLOQ, for which within ±20% was acceptable. In the case of precision, the acceptance criterion was a CV <15%, except at LLOQ, for which a CV <20% was acceptable. The extraction recovery was determined by comparing the peak areas of each analyte in two extracts: one was prepared from matrix spiked with the standard; the other was authentic standard dissolved in the mobile phase. Recovery was assessed at three concentrations: 1, 10, 100 ng/mL, with three determinations per concentration. The matrix effect was determined by comparing the peak area of each analyte in the extract prepared by adding the standard to the extract from blank matrix with the peak area of the authentic standard dissolved in the mobile phase. The matrix effect was assessed at the concentrations 100 ng/mL.

### 2.9 Statistics

Results were processed using the Excel 2013 Quick Analysis Tool (Microsoft, St. Redmond, WA, USA).

## 3. Results

**Fig 1:**
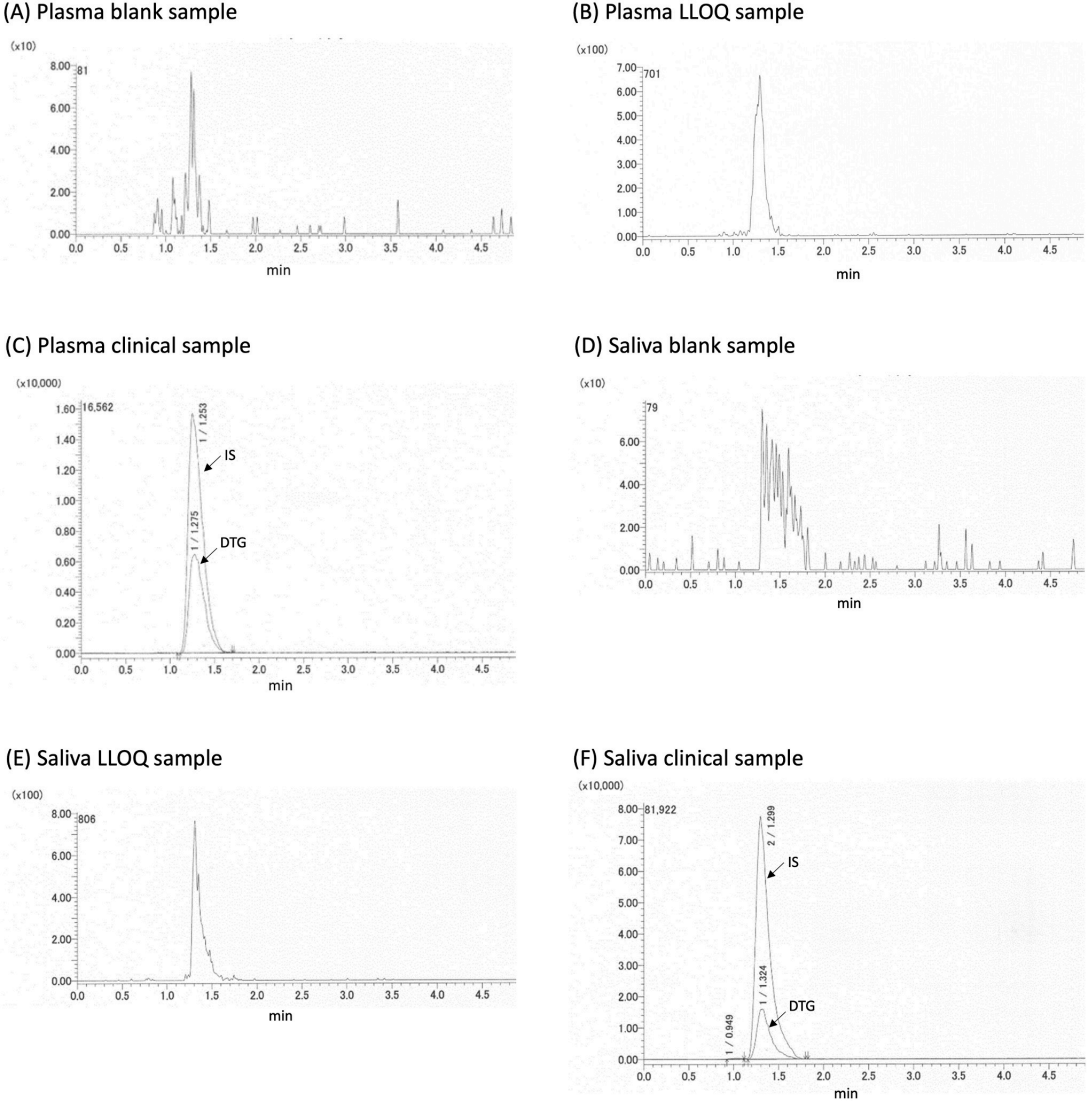
Chromatograms of a blank sample, a LLOQ sample, and a clinical sample for plasma and saliva.

First, we validated the quantification method of DTG in plasma and saliva. The LLOQ was 0.5 ng/mL according to the criteria (see Materials and methods). Calibration curves were linear in the range of 0.5–100 ng/mL with precision of <14.4% and accuracy within ±14.7. The R2 values were 0.9998 for plasma and saliva. Chromatograms of a blank sample, a LLOQ sample, and a clinical sample for plasma and saliva are shown in Fig 1A–1F. No peak was detected in analyses of blank plasma and saliva samples, suggesting the specificity for DTG. The results obtained for accuracy and precision, recovery, matrix effect and selectivity in both plasma and saliva are presented in Table 1 showing acceptable criteria. We obtained only 100 ng/mL data for matrix effect and plasma result was 117.1%, but the range of 70–120% has been determined as apparent matrix effect in generally [12, 13]. Therefore, we resulted this was not a significant effect. For stability, we didn’t demonstrate that, but Penchala et al. [14] showed DTG stability in plasma keeping on bench-top (16 h), after re-injection (48 h), heat inactivation and 3 cycles of freeze-thaw, and long term stability (10 months). Though DTG stability in saliva has not been evaluated, at least four antiretroviral drugs stability was shown in our previous study [11]. Taken together, a sensitive and robust LC–MS/MS method has been developed and validated for the accurate measurement of DTG in human plasma and saliva.

**Table 1:** Accuracy and precision, recovery, matrix effect and selectivity of the DTG quantification method.

	Sample	Plasma		Saliva	
Accuracy and Precision		Accuracy (%)	Precision (%)	Accuracy (%)	Precision (%)
	LLOQ (0.5 ng/mL)	19.5	17.0	16.8	8.0
	Low QC (1 ng/mL)	−3.2	7.4	5.0	14.4
	Middle QC (5 ng/mL)	2.5	6.6	−14.7	11.7
	High QC (50 ng/mL)	3.6	8.7	−8.5	8.8
	ULOQ (100 ng/mL)	−1.9	6.3	2.8	5.6
Recovery ± SD (%)	1 ng/mL	108.1 ± 0.6		112.3 ± 4.5	
	10 ng/mL	88.2 ± 2.3		85.8 ± 2.5	
	100 ng/mL	110.9 ± 2.5		112.8 ± 1.8	
Matrix effect ± SD (%)	100 ng/mL	117.1 ± 6.5		112.5 ± 2.5	
Selectivity		Measured conc. (ng/mL)	CV (%)	Measured conc. (ng/mL)	CV (%)
	A	0.52	11.9	0.41	1.4
	B	0.51	1.4	0.43	1.4
	C	0.58	1.4	0.52	2.9
	D	0.44	0.2	0.51	2.1
	E	0.41	9.6	0.58	4.3
	F	0.49	10.6	0.52	3.5

Accuracy and precision were calculated from data of five assays.

A total of 17 pairs of blood and saliva samples collected from 15 HIV-1-infected patients (11 men, 4 women) treated with the regimen containing DTG for more than one month were analyzed. The times from the last drug intake to sample collection was 13.5±6.0 h (mean±SD) for blood and 14.3±5.8 h for saliva. Median age (range) was 46 (35–70) years; median CD4 cells/mm^3^, 510 (292–1104); the percentage of patients with HIV viral load of <20 copies/mL, 88%; median treatment period with DTG, 19 (1–45) months.

**Fig 2:**
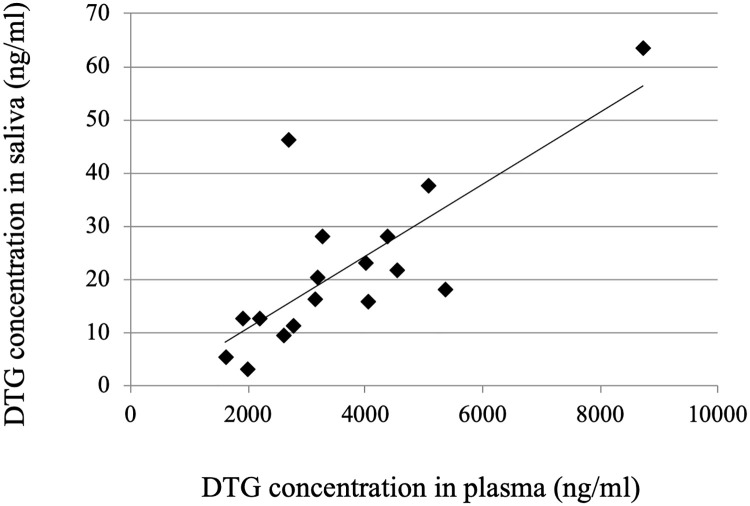
Relationship between the DTG concentrations in paired samples of plasma and saliva.

The relationship between the DTG concentrations in plasma and saliva is shown in Fig 2. The median DTG concentrations in plasma and saliva were, respectively, 3161 ng/mL (range, 1608–8724 ng/mL) and 17.7 ng/mL (2.9–63.3 ng/mL). There is a significant correlation between the DTG concentrations in the two compartments (Pearson’s correlation coefficient *r* = 0.76, *p* < 0.001). The median ratio of the DTG concentration in saliva to that in plasma was 0.0056 (range 0.0015–0.0171).

**Fig 3:**
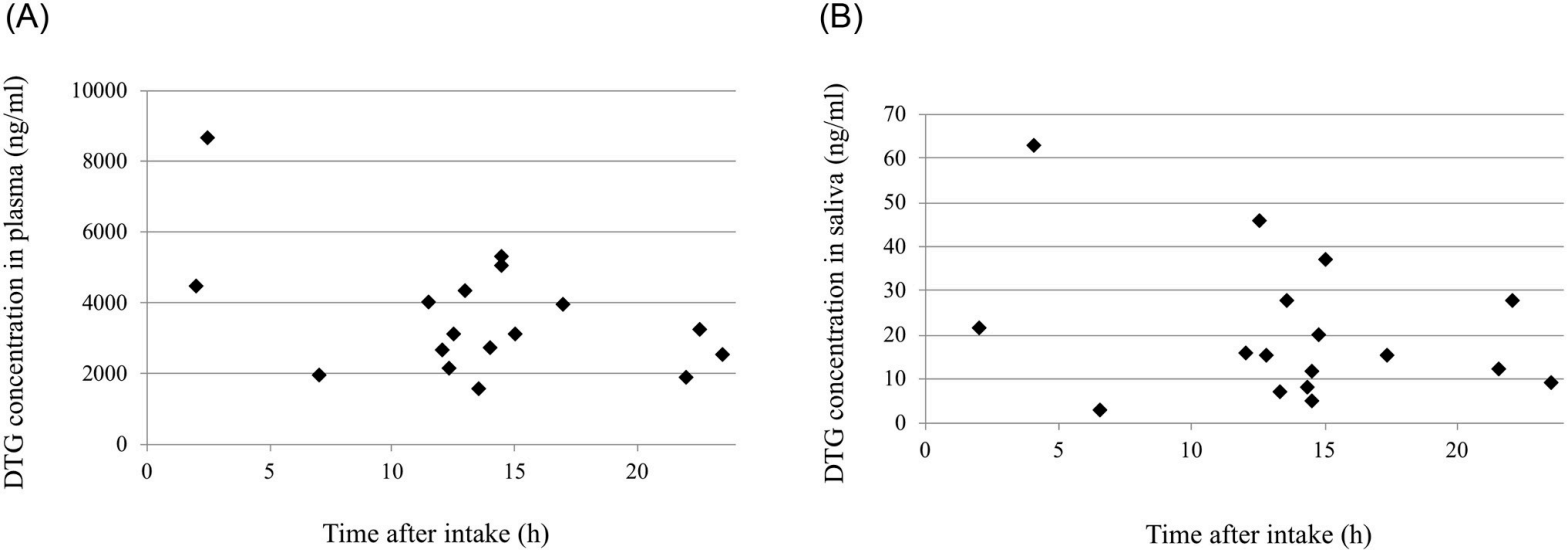
The time after oral administration and drug concentrations in plasma (A) and saliva (B).

DTG concentrations of 17 pairs of plasma and saliva samples from 15 different subjects were plotted against the time from intake to sampling (Fig 3A and 3B). The DTG concentrations did not show a typical pharmacokinetic curve although only plots near t_max_ of 2.0 h [15] were relatively high (Fig 3A and 3B). This may reflect inter-individual differences in pharmacokinetics. It was reported that steady-state C_max_ and C_trough_ of DTG were 3.69 μg/mL and 1.10 μg/mL, respectively [14]; some of DTG concentrations determined in this study were a little higher than the C_max_.

## 4. Discussion

We showed a strong correlation between the DTG concentrations in plasma and saliva using a validated LC-MS/MS method, suggesting the feasibility of using saliva for TDM as an alternative to blood. However, the ratio of the two concentrations varied among individuals (range, 0.0015–0.0171), and the correlation coefficient (0.76) was lower than those of abacavir (0.94), darunavir (0.88), and raltegravir (0.93), which have been reported in our previous study [4]. Therefore, the plasma-to-saliva ratio may have to be determined for an individual patient beforehand and used to normalize saliva concentrations, thus making this a suitable matrix for TDM of DTG.

Dolutegravir is highly lipophilic and bound to plasma protein including albumin and α_1_-acid glycoprotein [15]. It has been reported that the median unbound fraction of DTG in plasma after 16 weeks of therapy is 0.70% [16], which is similar to the median ratio of DTG saliva concentration to plasma concentration found in this study. This finding suggests that the secretion of DTG into saliva is governed mainly by passive transcellular diffusion, rather than transcellular active transport, of the unbound drug in plasma [17]. Similar observations were made in other antiretroviral drugs such as nevirapine [18], abacavir, darunavir, and raltegravir [4].

Transmission of HIV through oral sex occurs at a low frequency [19, 20]. The saliva concentrations of DTG of all subjects in this study were above the *in vitro* 50% inhibitory concentration (IC_50_) for wild-type HIV (1.1 ng/mL) [21]. The DTG in saliva can be considered to be mostly unbound because the concentrations of albumin and α_1_-acid glycoprotein in saliva were extremely low compared with those in plasma [22, 23]. The use of tenofovir disoproxil fumarate/emtricitabine is recommended for pre-exposure prophylaxis (PrEP) for HIV infection in high-risk individuals [24]. A sub-study of the ANRS IPERGAY showed that the emtricitabine level in saliva was above the IC_50_, while the tenofovir level was suboptimal, suggesting it is little protective against oral HIV transmission. Our data suggest that DTG may be an alternative drug for PrEP even for oral transmission.

Our study has several limitations. The sample size was small, only 17, and the time between intake and sampling was varied considerably from person to person. Saliva samples were collected after plasma samples within 1 hour, which may have underestimated the ratio of saliva-to-plasma DTG concentrations based on its pharmacokinetics. In addition, data in this study is insufficient to evaluate the usefulness of saliva for TDM because the number of samples for each subject was only 1 or 2. In the study on saliva as an alternative matrix for pharmacokinetics, it should be noted that the saliva concentration of xenobiotics depends on not only plasma concentration but also the patient’s health condition, diet, tooth or gingiva condition and so on, as discussed previously [25].

In conclusion, our results are supportive of the possibility of using saliva as an easily accessible alternative for TDM of DTG. Also, the DTG concentration in saliva reflects the pharmacologically active drug concentration in plasma and a regimen containing DTG may be a candidate for PrEP against oral HIV transmission.
